# Supplementary material for: Fgf3 is crucial for the generation of monoaminergic cerebrospinal fluid contacting cells in zebrafish
Source: Biol Open. 2019 Jun 15;8(6):bio040683. doi: 10.1242/bio.040683 (PMC6602327; doi:10.1242/bio.040683)
Supplement: Supplementary information [file biolopen-8-040683-s1.pdf]

Supplementary data

Table S1. Target sites, oligos and PCR primers for *fgf3* CRISPR/Cas9 approach.

	***fgf3* exon 1 (5'-3')**	***fgf3* exon 2 (5'-3')**
**Target site**	GGGGTTTACGAGCACCTCGG	GGCAATCAAGGGACTGTTTT
**Oligo 1**	TAGGGGTTTACGAGCACCTCGG	TAGGCAATCAAGGGACTGTTTT
**Oligo 2**	AAACCCGAGGTGCTCGTAAACC	AAACAAAACAGTCCCTTGATTG
**Primer fwd**	AGCTTCTTGGATCCGAGTTTGG	ATACGCTTTCAGACAAGGCAAT
**Primer rev**	GATCCGTCTATTTTCCCGTTCG	CCCGACGTGACATAACACTTAC

Table S2. RNA probes used for whole mount RNA *in situ* hybridisation.

Gene name	Gene symbol	Reference
arginine vasopressin	*avp*	Eaton et al., 2008
cortistatin	*cort*	Devos et al., 2002
dual specificity phosphatase 1	*dusp1*	this study
dual specificity phosphatase 6	*dusp6*	Tsang et al., 2004
ets variant 5b	*etv5b*	Münchberg et al., 1999
fibroblast growth factor 3	*fgf3*	Kiefer et al., 1996
NK2 homeobox 4b	*nkx2.4b*	Rohr and Concha, 2000
oxytocin	*oxt*	Unger and Glasgow, 2003
tyrosine hydroxylase 2	*th2*	Yamamoto et al., 2010

Table S3. Overview of counting of monoaminergic and neuroendocrine cells, and of measurements of the *nkx2.4b* expressing hypothalamic area in embryos after *fgf3* impairment.

	**marker**	**5–HT**		**TH1**				***th2***	***oxt***	***avp***	***cort***	***nkx2.4b***						**Fish length**	**phH3**	**BrdU**	**cCasp3**
	**region**	**i./p.**		**DC 4/5/6**		**DC 7**		**DC 7**				**ventral**			**lateral**						
	**hpf**	72	96	72	96	72	96	96	72	72	72	36	48	72	36	48	72	72	36	36	36
***fgf3* morpholino**	**UC**	64 ±11	98 ±13	59 ±4	67 ±6	20 ±5	47 ±9	40 ±4	27.5 ±5	57 ±4	127.5 ±9.5	37950 ±3352	37920 ±3250	56190 ±4836	53270 ±3068	38100 ±6846	39410 ±5779	2464000 ±45710	25.5 ±7	109 ±16.5	0.5 ±0.5
	**n**	13	15	13	15	13	17	9	28	28	31	27	26	40	27	23	24	21	12		5
	**MO**	35 ±6	66 ±7	52 ±2	62 ±6	6 ±4	31 ±3	27 ±7	27.5 ±7	51 ±6	128 ±14	30840 ±3067	32010 ±3085	48070 ±3066	42600 ±4084	30730 ±4183	37850 ±3597	2463000 ±78618	21 ±4.5	84.5 ±13.5	10±1
	**n**	19	17	19	17	19	17	11	24	20	25	19	22	24	18	24	24	13	12		4
	**p**	4.56 e^{-06}	1.97 e^{-06}	0.191	0.1903	1.24 e^{-05}	9.53 e^{-06}	0.0011	n.s. (0.3651)	0.0003	n.s. (0.5653)	1.08 e^{-06}	0.0001	6.83 e^{-09}	4.383 e^{-06}	n.s. (0.2)	n.s. (0.3137)	n.s. (0.8097)	n.s. (0.862)	0.0408	0.0016
***fgf3*^{t24152} mutant**	**+/+**	60 ±22	-	58 ±6	-	11 ±4	-	-	27 ±4	61 ±7	86 ±10.5	28780 ±1832	28110 ±2861	42600 ±811	-	-	-	-	-	-	-
	**n**	23	-	25	-	25	-	-	14	18	20	12	17	10	-	-	-	-	-	-	-
	**+/-**	59.5 ±14	-	55 ±7	-	12 ±4	-	-	26 ±3	59.5 ±5	90 ±11	28310 ±2240	26390 ±1932	40870 ±1624	-	-	-	-	-	-	-
	**n**	46	-	51	-	51	-	-	38	26	41	36	34	18	-	-	-	-	-	-	-
	**-/-**	50 ±14.5	-	52.5 ±8.5	-	7.5 ±4.5	-	-	31 ±3	51 ±4	97 ±9	26950 ±1370	25160 ±1306	36670 ±1842	-	-	-	-	-	-	-
	**n**	18	-	20	-	20	-	-	19	15	16	11	20	19	-	-	-	-	-	-	-
	**p**	0.04	-	n.s. (0.35)	-	0.0097	-	-	0.0169	0.0024	n.s. (0.492)	0.0757	0.038	3.7 e^{-08}	-	-	-	-	-	-	-
**CRISPR/Cas9**	**UC**	75 ±12	-	64.5 ±3	-	28.5 ±4.5	-	-	-	-	-	-	-	-	-	-	-	-	-	-	-
	**n**	12	-	12	-	12	-	-	-	-	-	-	-	-	-	-	-	-	-	-	-
	**C9C**	65.5 ±6	-	70.5 ±5.5	-	33 ±3.5	-	-	-	-	-	-	-	-	-	-	-	-	-	-	-
	**n**	16	-	16	-	16	-	-	-	-	-	-	-	-	-	-	-	-	-	-	-
	**CR**	38 ±16	-	62.5 ±5.5	-	18.5 ±6	-	-	-	-	-	-	-	-	-	-	-	-	-	-	-
	**n**	22	-	12	-	12	-	-	-	-	-	-	-	-	-	-	-	-	-	-	-
	**p**	0.0007	-	n.s. (0.0917)	-	1.31 e^{-07}	-	-	-	-	-	-	-	-	-	-	-	-	-	-	-

Cell numbers and area sizes (pixels) displayed as median±MAD. p = p-values determined by two-sample t-test, Mann-Whitney-U test, one-way ANOVA, or Kruskal-Wallis test. n = number of analysed individuals. UC, uninjected control; MO, morphant; C9C, Cas9 only injected control; CR, CRISPR/Cas9 injected

Table S4: List of Fgf-signalling genes selected for expression analysis in the hypothalamus by RNA sequencing.

***pea3*-family, *fgf, fgfr* genes:**

etv1	*fgf6a*	*fgf12a*	*fgf20b*
etv4	*fgf6b*	*fgf12b*	*fgf21*
etv5a	*fgf7*	*fgf13a*	*fgf22*
etv5b	*fgf8a*	*fgf13b*	*fgf23*
fgf1a	*fgf8b*	*fgf14*	*fgf24*
fgf1b	*fgf9*	*fgf16*	*fgfr1a*
fgf2	*fgf10a*	*fgf18a*	*fgfr1b*
fgf3	*fgf10b*	*fgf18b*	*fgfr2*
fgf4	*fgf11a*	*fgf19*	*fgfr3*
fgf5	*fgf11b*	*fgf20a*	*fgfr4*

Fgf-signalling component genes:

araf	*dusp3a*	*flrt3*	*kras*	*nf1b*	*rras2*	*tnip1*
braf	*dusp3b*	*frs2a*	*lamtor3*	*nras*	*shc1*	*tnip2*
cnpy1	*dusp4*	*frs2b*	*map2k1*	*ptpn5*	*shc2*	
cnpy2	*dusp5*	*frs3*	*map2k2a*	*raf1a*	*sos1*	
cnpy3	*dusp6*	*gab1*	*map2k2b*	*raf1b*	*sos2*	
cnpy4	*dusp7*	*grb2a*	*mapk1*	*rasa1a*	*spry1*	
dusp1	*dusp8a*	*grb2b*	*mapk3*	*rasa1b*	*spry2*	
dusp10	*flrt1a*	*hrasa*	*MRAS*	*rasa3*	*spry4*	
dusp16	*flrt1b*	*hrasb*	*mras*	*rasa4*	*syngap1a*	
dusp2	*flrt2*	*kl*	*nf1a*	*rras*	*syngap1b*	

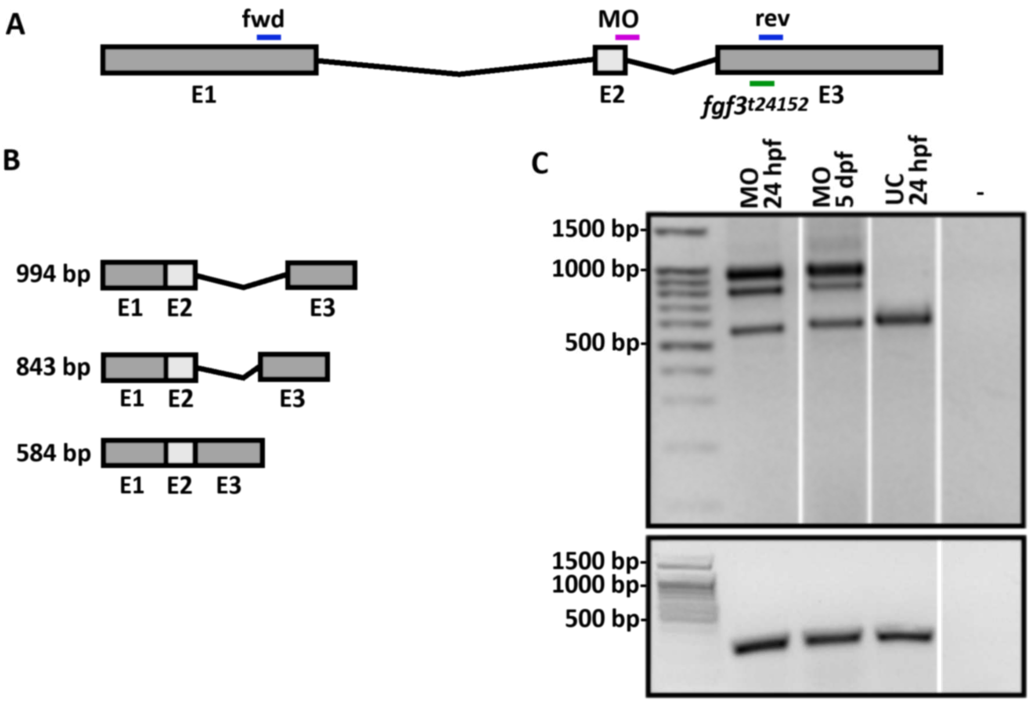

D

```
Fgf3 WT        1 MVIILLLLLLSFLDPSLEESLAPRLTRTPRAPCARGQACDPRQRRDAGGR  50
Fgf3t24152     1 MVIILLLLLLSFLDPSLEESLAPRLTRTPRAPCARGQACDPRQRRDAGGR  50
Fgf3 MO        1 MVIILLLLLLSFLDPSLEESLAPRLTRTPRAPCARGQACDPRQRRDAGGR  50
                 **************************************************

Fgf3 WT       51 GGVYEHLGGAPRRRKLYCATKYHLQIHPNGKIDGSLEENNPLSILEITAV 100
Fgf3t24152    51 GGVYEHLGGAPRRRKLYCATKYHLQIHPNGKIDGSLEENNPLSILEITAV 100
Fgf3 MO       51 GGVYEHLGGAPRRRKLYCATKYHLQIHPNGKIDGSLEENNPLSILEITAV 100
                 **************************************************

Fgf3 WT      101 DVGVVAIKGLFSGRYLAMNEKGRLYASE-VF-NRECEFLERIHELGYNTY 148
Fgf3t24152   101 DVGVVAIKGLFSGRYLAMNEKGRLYASE-VF-NRECEFLERIHELGYNTY 148
Fgf3 MO      101 DVGVVAIKGLFSGRYLAMNEKGRLYASVSVMSRRENQVAEILLAVGSDND 150
                 ***************************  *: .** :. * :   :* :.

Fgf3 WT      149 ASRHHATTQPPPTGSGIGGSKRRASSKRQWYVSINGKGRPRRGFKTRSTD 198
Fgf3t24152   149 ASRHHATTQPPPTGSGIGGSKRRASSKRQ*-------------------- 178
Fgf3 MO      151 *-------------------------------------------------

Fgf3 WT      199 KASLFLPRVLANKDHEMVRKLRESQRHHTGSHRAPVGRAERRRRRHRGSK 248
Fgf3t24152       --------------------------------------------------
Fgf3 MO          --------------------------------------------------

Fgf3 WT      249 GHNRRADI                                          256
Fgf3t24152       --------
Fgf3 MO          --------
```

Fig. S1. *fgf3* morpholino (MO) knock-down efficiency and amino acid sequence alignment of wildtype (WT), mutant (*fgf3*^{t24152}) and morphant Fgf3. (A) Scheme of *fgf3* gene and locations of splice MO target site (magenta), as well as forward (fwd) and reverse (rev) primers (blue) used for detection of morphant splice forms, and point mutation *fgf3*^{t24152} (green). **(B)** Schematic representation of splice products detected by RT-PCR after MO injections. **(C)** RT-PCR results of morphants at 24 hpf and 5 dpf, uninjected control (UC) siblings at 24 hpf and water control (-) for *fgf3* (upper gel) and *β-actin* (lower gel). Additional RT-PCR products in *fgf3* morphants at 843 and 994 bp corresponded to a partial intron 2 inclusion due to a cryptic splice site or a complete inclusion of intron 2, respectively. **(D)** Amino acid sequence alignment of WT, *fgf3*^{t24152} mutant and morphant Fgf3. The *fgf3*^{t24152} mutation results in a premature stop after amino acid 177 (Q), thus, 69% of the WT protein sequence remains intact. After MO application, both splice forms of *fgf3* generate a nonsense sequence after amino acid 127 (S, magenta) and a stop after amino acid 150 (D), thus, 50% of the WT protein sequence is intact.

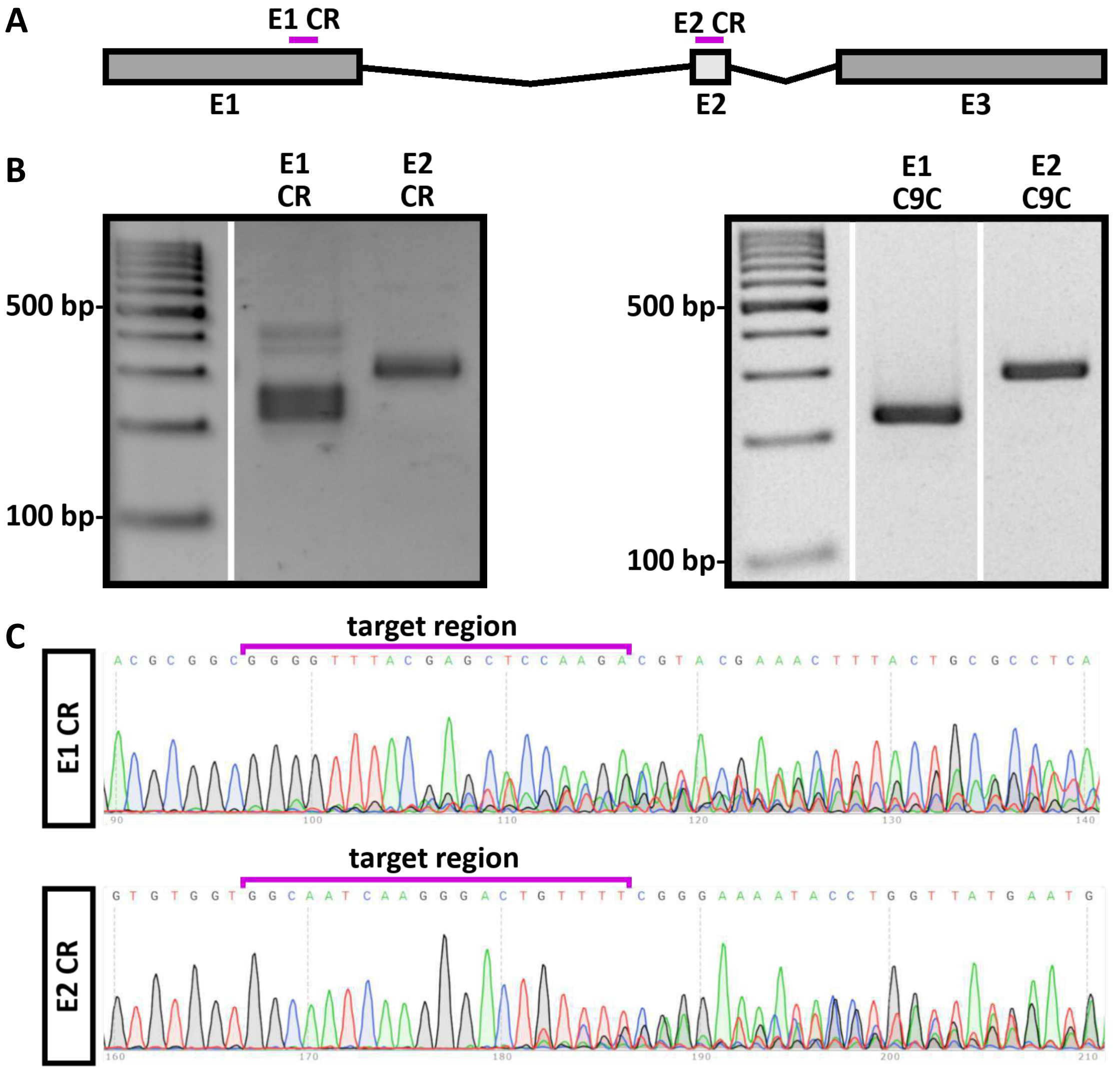

Fig. S2. Validation of fgf3 CRISPR/Cas9. (A) Scheme of *fgf3* gene with location of gRNA target region in exon 1 (E1 CR) and exon 2 (E2 CR) highlighted in magenta. **(B)** Examples of genotyping results from one 72 hpf embryo injected with both gRNAs together with Cas9 (left gel) and one 72 hpf control embryo injected with Cas9 only (C9C) (right gel). Note multiple bands for E1 CR and E2 CR gRNA in CRISPR/Cas9 injected embryo. The control embryo shows single wildtype bands (E1 C9C: 224 bp, E2 C9C: 289 bp) as a result of PCR amplification of E1 and E2 target regions. **(C)** DNA sequencing traces of genotyping PCR of CRISPR/Cas9 injected embryo (same specimen as for left gel in B) for E1 and E2 gRNA target regions revealed multiple traces due to indel mutations.

Biology Open • Supplementary information

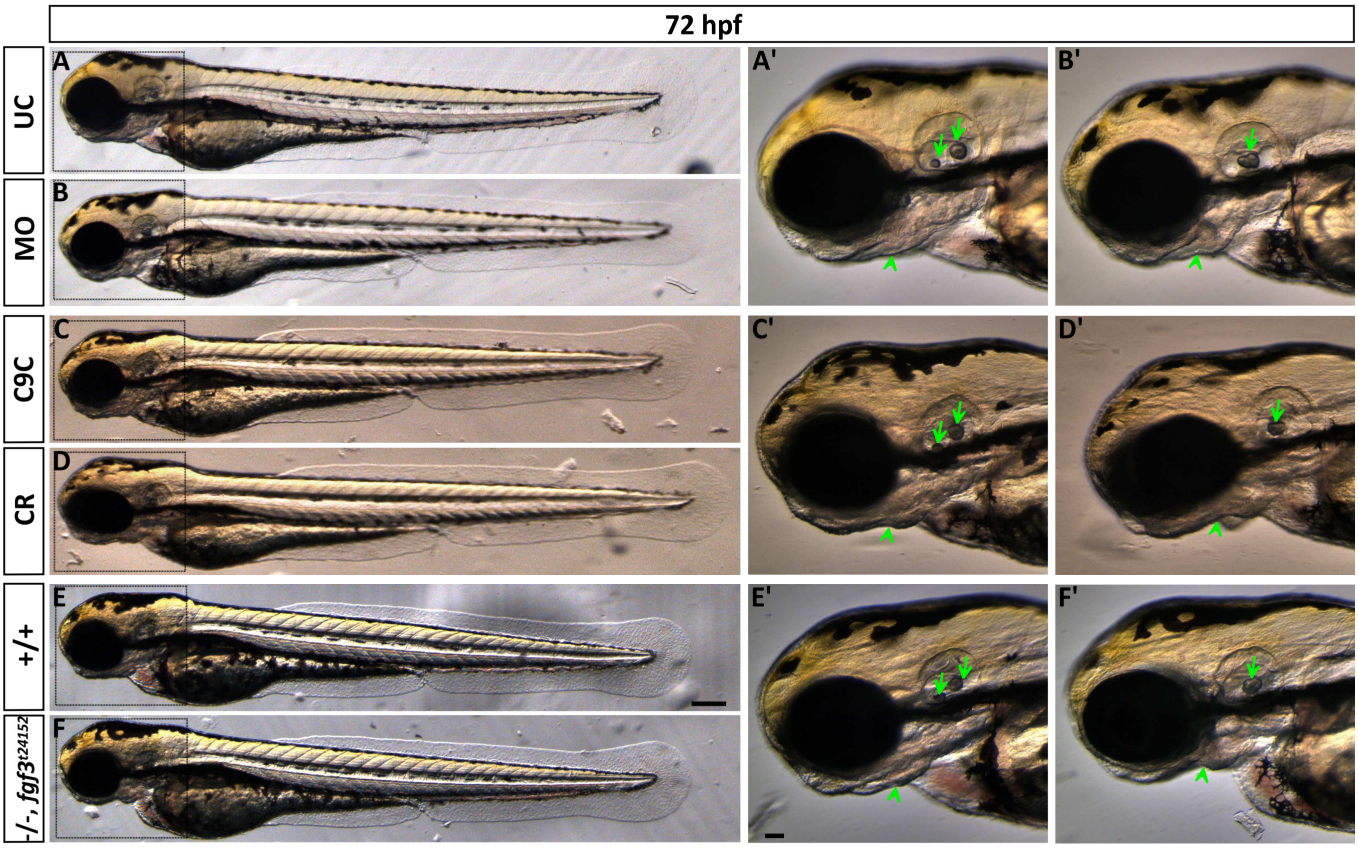

Fig. S3. Live images showing morphology of 72 hpf embryos after *fgf3* impairment with characteristic ear and craniofacial malformations. (A,B) Uninjected control (UC) and *fgf3* morpholino injected (MO) siblings. **(C,D)** Cas9 injected control (C9C) and *fgf3* CRISPR/Cas9 injected (CR) siblings. **(E,F)** Wildtype (+/+) and homozygous *fgf3t24152* mutant (-/-) siblings. Boxes indicate magnified area shown in **A'-F'**. After *fgf3* impairment the two otoliths fuse (arrow) and the lower jaw bones are malformed (arrow heads). Lateral views, anterior to the left. Scale bar in **E**, 100 µm; in **E'**, 50 µm.

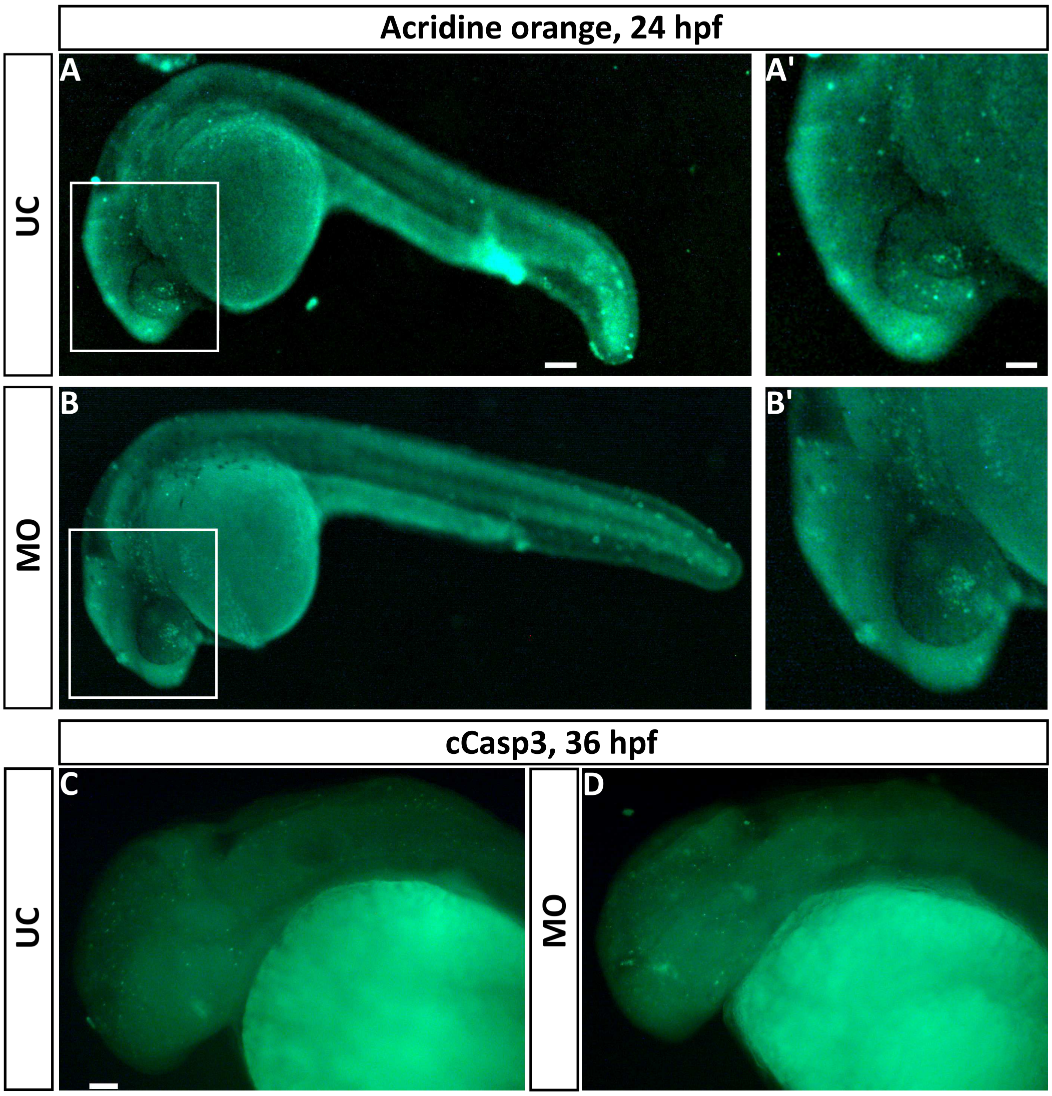

Fig. S4. Fluorescence pictures showing cell death in *fgf3* morphants. (A,B) Live images of acridine orange stained uninjected control (UC) and morphant (MO) siblings at 24 hpf. **A'** and **B'** are high magnifications of boxed areas in **A** and **B**. **(C,D)** Cleaved caspase 3 (cCasp3) immuno stained control and morphant siblings at 36 hpf. Lateral views, anterior to the left. Scale bar in **A**, 100 µm; in **A'** and **C** 50 µm.

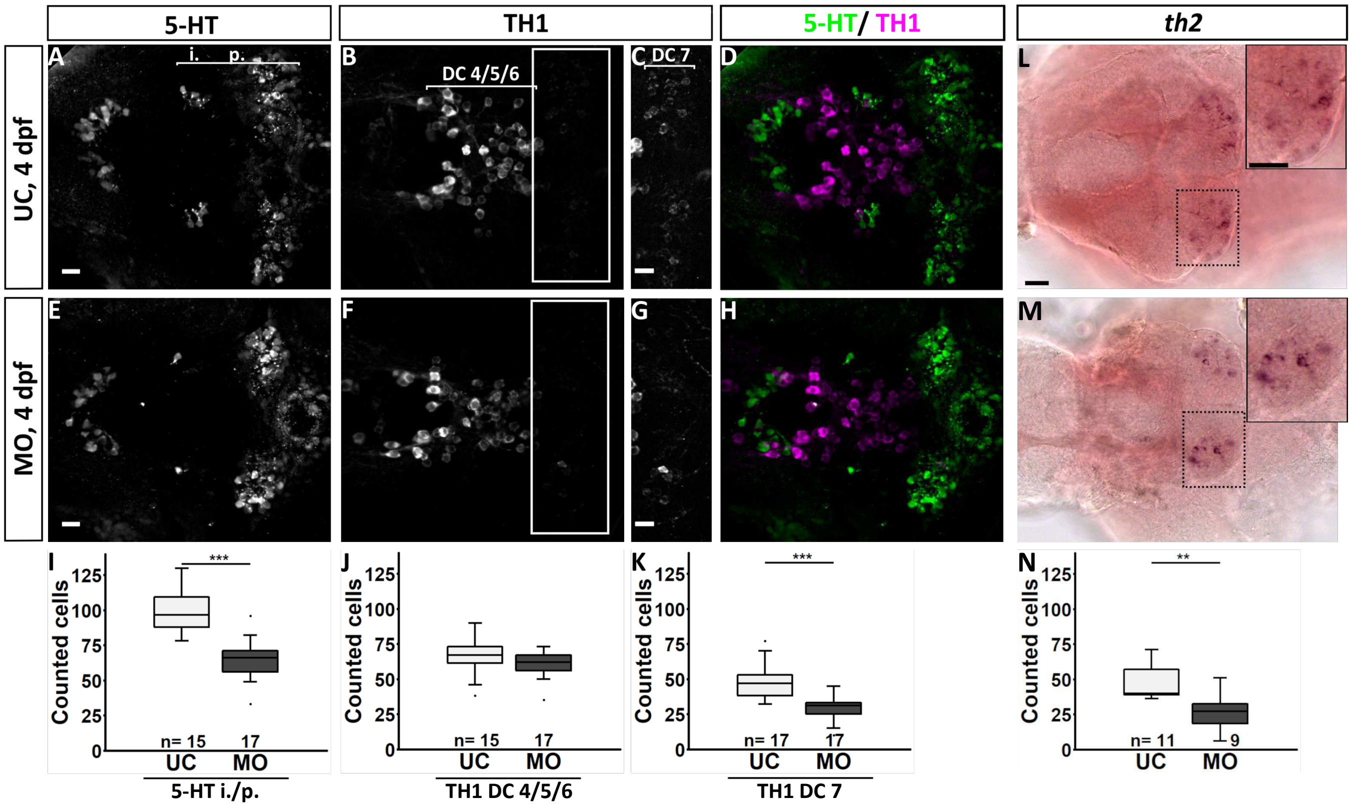

Fig. S5. Quantification of the number of serotonergic cells in the intermediate (i.)/posterior (p.) clusters and of dopaminergic cells in the DC 4/5/6 and DC 7 clusters in the hypothalamus of *fgf3* morphants at 4 dpf. (A-H) Confocal maximum intensity projections from uninjected control (UC) and morpholino injected (MO) siblings immuno stained for 5-HT (green) and TH1 (magenta) shown as single channels and merged. **C** and **G** show boxed areas in **B** and **F**, respectively, with adjusted brightness and contrast to reveal the faint TH1 immunoreactive cells of the DC 7 cluster. **(L, M)** Light microscopic pictures of *fgf3* morphants and uninjected control siblings processed for RNA *in situ* hybridisation for *th2* expressed by dopaminergic cells intermingled with TH1 positive cells in the DC 7 cluster. Insets show high magnifications of boxed areas. Ventral views, anterior to the left. Scale bars in **A,E,C,G**, 10 µm; in **L**, 30 µm. **(I-K, N)** Quantifications of 5-HT, TH1 and *th2* positive cells in control and morphant siblings. The number of serotonergic cells was counted in the i./p. clusters as indicated by the line in **A**. The number of dopaminergic (TH1 and *th2*) cells was counted in the DC 4/5/6 and DC 7 clusters as indicated by the lines in **B** and **C**, respectively. Tukey boxplots showing median, 25-75% percentile, IQR whiskers and outliers. n = number of analysed individuals.

Biology Open • Supplementary information

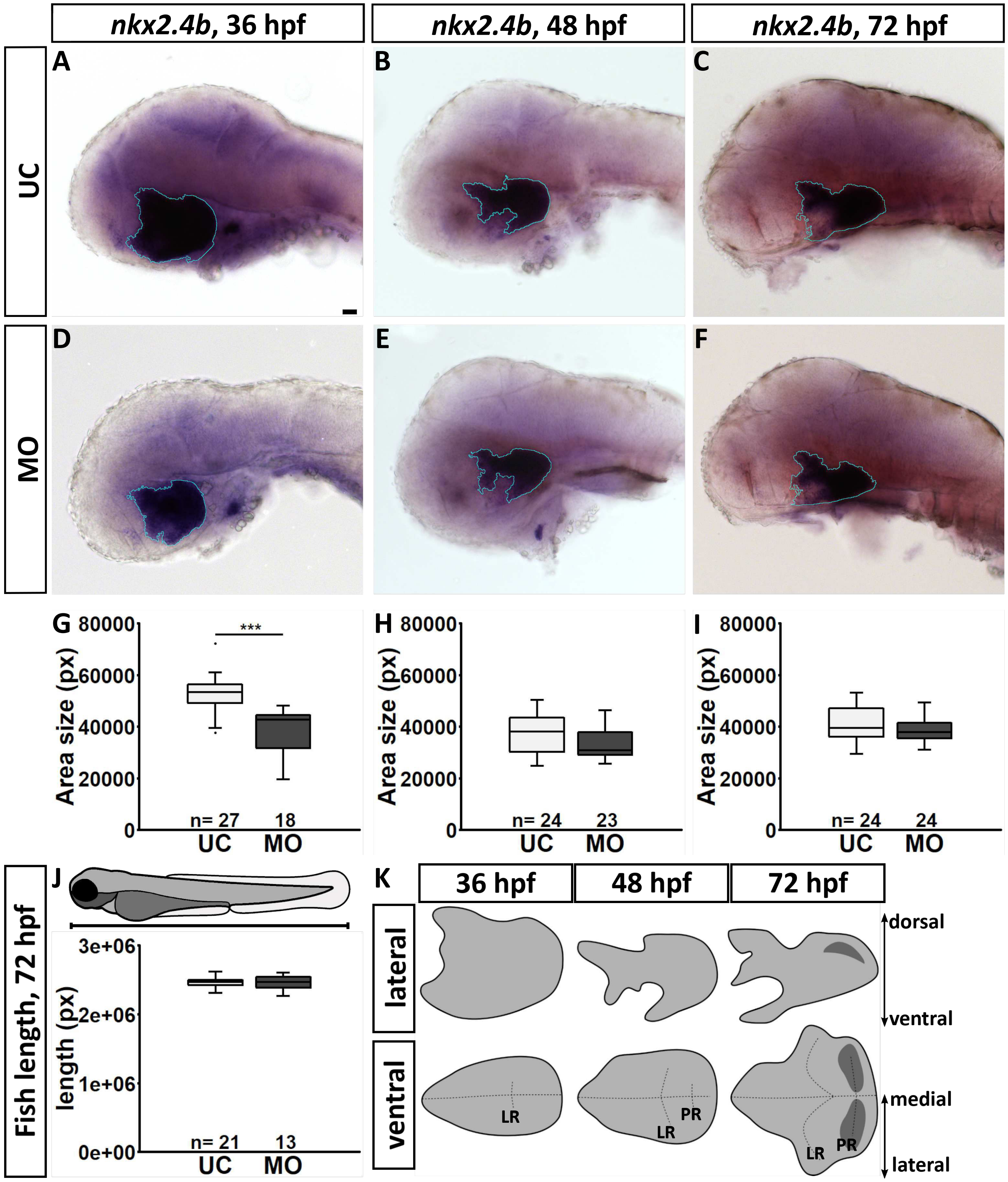

Fig. S6. The shape of the hypothalamic *nkx2.4b* domain is altered in *fgf3* morphants. (A-F) Light microscopic pictures showing expression of *nkx2.4b* in *fgf3* morphants (MO) and uninjected control siblings (UC) at 36, 48 and 72 hpf visualised by RNA *in situ* hybridisation. Outlines of semi-automated measurement of hypothalamic area are highlighted in blue. Lateral views, anterior to the left. Scale bar = 30 µm. **(G-I)** Area measurements (pixels) in *fgf3* morphants and control siblings of the *nkx2.4b* domain at 36, 48 and 72 hpf. **(J)** Total length measurements of *fgf3* morphants and control siblings at 72 hpf. Tukey boxplots showing median, 25-75% percentile, IQR whiskers and outliers. n= number of analysed individuals. **(K)** Scheme illustrating the lateral and ventral silhouette of the *nkx2.4b* expressing hypothalamic domain at 36, 48 and 72 hpf. Dark grey indicates location of monoaminergic populations around the posterior recess (PR). Dashed lines show hypothalamic ventricular system with the (LR) lateral recess and the PR.

Biology Open • Supplementary information

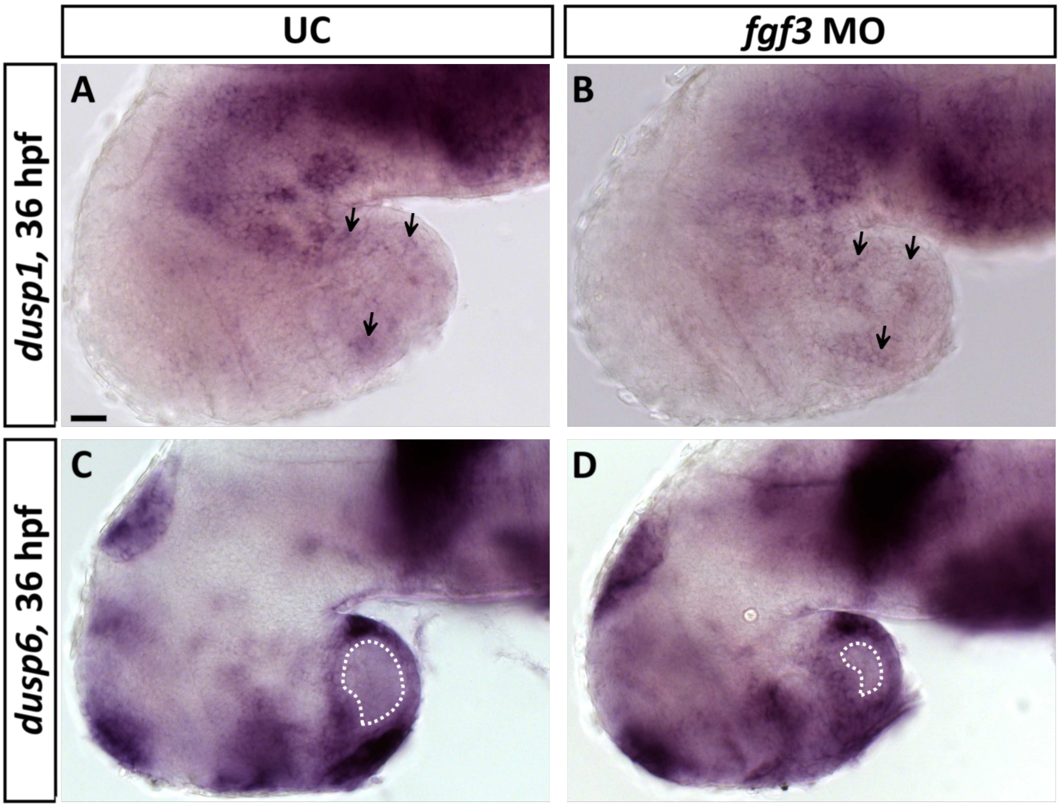

Fig. S7. *fgf3* morphants exhibit mild alterations in expression of some Fgf-signalling targets. (A-D) Light microscopic pictures of uninjected control (UC) and *fgf3* morphant (MO) siblings processed for RNA *in situ* hybridisation for *dusp1* and *dusp6* at 36 hpf. Notably, the hypothalamic *dusp1* expression is weaker in morphants compared to controls in hypothalamus (arrows) as well as in other brain regions (arrows). For *dusp6* the expression intensity is similar, but the size of the *dusp6* negative domain in posterior hypothalamus is reduced (dashed line). Lateral views, anterior to the left. Scale bar = 30 µm.

References

Devos, N., Deflorian, G., Biemar, F., Bortolussi, M., Martial, J. A., Peers, B., Argenton, F. (2002). Differential expression of two somatostatin genes during zebrafish embryonic development. *Mechanisms of Development*. **1–2**, 133–137.

Eaton, J. L., Holmqvist, B., Glasgow, E. (2008). Ontogeny of vasotocin-expressing cells in zebrafish: selective requirement for the transcriptional regulators orthopedia and single-minded 1 in the preoptic area. *Dev. Dyn.* **4**, 995–1005.

Kiefer, P., Strähle, U., Dickson, C. (1996). The zebrafish Fgf-3 gene: cDNA sequence, transcript structure and genomic organization. *Gene*. **2**, 211–215.

Münchberg, S. R., Ober, E. A., Steinbeisser, H. (1999). Expression of the Ets transcription factors erm and pea3 in early zebrafish development. *Mechanisms of Development*. **2**, 233–236.

Rohr, K. B., Concha, M. L. (2000). Expression of nk2.1a during early development of the thyroid gland in zebrafish. *Mechanisms of Development*. **1-2**, 267–270.

Tsang, M., Maegawa, S., Kiang, A., Habas, R., Weinberg, E., Dawid, I. B. (2004). A role for MKP3 in axial patterning of the zebrafish embryo. *Development*. **12**, 2769–2779.

Unger, J. L., Glasgow, E. (2003). Expression of isotocin-neurophysin mRNA in developing zebrafish. *Gene Expression Patterns*. **1**, 105–108.

Yamamoto, K., Ruuskanen, J. O., Wullimann, M. F., Vernier, P. (2010). Two tyrosine hydroxylase genes in vertebrates New dopaminergic territories revealed in the zebrafish brain. *Mol Cell Neurosci*. **4**, 394–402.

Biology Open • Supplementary information
